# Nutritional Control of DNA Replication Initiation through the Proteolysis and Regulated Translation of DnaA

**DOI:** 10.1371/journal.pgen.1005342

**Published:** 2015-07-02

**Authors:** David J. Leslie, Christian Heinen, Frederic D. Schramm, Marietta Thüring, Christopher D. Aakre, Sean M. Murray, Michael T. Laub, Kristina Jonas

**Affiliations:** 1 LOEWE Center for Synthetic Microbiology, Philipps University Marburg, Marburg, Germany; 2 Max Planck Institute for Terrestrial Microbiology, Marburg, Germany; 3 Department of Biology, Massachusetts Institute of Technology, Cambridge, Massachusetts, United States of America; 4 Howard Hughes Medical Institute, Massachusetts Institute of Technology, Cambridge, Massachusetts, United States of America; 5 Department of Biology, Philipps University Marburg, Marburg, Germany; Agency for Science, Technology and Research, SINGAPORE

## Abstract

Bacteria can arrest their own growth and proliferation upon nutrient depletion and under various stressful conditions to ensure their survival. However, the molecular mechanisms responsible for suppressing growth and arresting the cell cycle under such conditions remain incompletely understood. Here, we identify post-transcriptional mechanisms that help enforce a cell-cycle arrest in *Caulobacter crescentus* following nutrient limitation and during entry into stationary phase by limiting the accumulation of DnaA, the conserved replication initiator protein. DnaA is rapidly degraded by the Lon protease following nutrient limitation. However, the rate of DnaA degradation is not significantly altered by changes in nutrient availability. Instead, we demonstrate that decreased nutrient availability downregulates *dnaA* translation by a mechanism involving the 5' untranslated leader region of the *dnaA* transcript; Lon-dependent proteolysis of DnaA then outpaces synthesis, leading to the elimination of DnaA and the arrest of DNA replication. Our results demonstrate how regulated translation and constitutive degradation provide cells a means of precisely and rapidly modulating the concentration of key regulatory proteins in response to environmental inputs.

## Author Summary

The duplication of genetic material is a prerequisite for cellular growth and proliferation. Under optimal growth conditions, when cells strive to grow and divide, DNA replication must be initiated with high frequency. However, under nutrient limiting conditions cells stop initiating DNA replication to ensure cellular integrity. Here, we identify mechanisms responsible for blocking DNA replication initiation under nutrient limitation in *Caulobacter crescentus*. In this bacterium nutrient limitation results in a strong downregulation of DnaA, the conserved replication initiator protein, which is required for DNA replication in nearly all bacteria. Our data demonstrate that the downregulation of DnaA depends on a reduction in DnaA synthesis in combination with fast degradation by the protease Lon. The changes in DnaA synthesis are mediated by a post-transcriptional mechanism, which adjusts DnaA translation in response to nutrient availability. The constitutively high rate of DnaA degradation then ensures the rapid clearance of the protein following the changes in translation. Our work exemplifies how regulated protein synthesis and fast degradation of key regulatory proteins allow for the precise and dynamic control of important cellular processes in response to environmental changes.

## Introduction

The ability of cells to arrest their growth and proliferation in response to nutrient depletion or stressful conditions is typically critical for their survival. Growth arrest requires global changes in protein synthesis leading to a decline in the production of cellular mass. Importantly, growth arrest also usually demands a concomitant cessation of cell cycle processes, including DNA replication. The mechanisms that modulate cell cycle progression following nutrient limitation remain poorly understood. A promising candidate for transducing information about nutritional status to the bacterial cell cycle is DnaA, the conserved replication initiator protein.

DnaA is a AAA+ ATPase required for replication initiation in most bacteria [1]. It directly binds to and unwinds the origin of replication and subsequently recruits replisome components. Several mechanisms have been reported in different bacteria that modulate DnaA activity to ensure the correct timing of DNA replication initiation [2]. One major mechanism, first elucidated in *Escherichia coli* and generally referred to as RIDA (regulatory inactivation of DnaA), involves ATP binding and hydrolysis [3,4]. Binding to ATP favors an active conformation that allows for the assembly of DnaA into an oligomeric structure that promotes duplex unwinding [1,5]. After initiation, ATP hydrolysis by DnaA can be stimulated (in *E*. *coli*) by the protein Hda bound to the DNA-loaded replicase clamp [6]. ATP hydrolysis inactivates DnaA and thereby helps prevent the re-initiation of DNA replication [4,7]. RIDA likely operates in other proteobacteria; additionally DnaA activity or its access to the origin can be regulated in some bacteria by interacting proteins or sequestration mechanisms [2].

Although there has been considerable progress in understanding how DnaA and replication initiation is coordinated with other cell cycle events, much less is known about how DnaA activity and DNA replication initiation are coordinated with changes in growth rate. One long-standing hypothesis posits that in steady-state growing cultures replication initiation is triggered at a constant cell-mass-to-origin ratio such that growth rate is intrinsically coupled to replication [8]. However, the precise mechanism responsible for this phenomenon remains unclear, and a number of studies have challenged this model [9–12]. Furthermore, it remains largely unexplored how DNA replication initiation is controlled during the transition from exponential growth to an arrested state, for example at entry into stationary phase, or during the onset of starvation upon nutrient limitation.

The α-proteobacterium *Caulobacter crescentus* is an important model system for understanding the bacterial cell cycle. *Caulobacter* cells are inherently asymmetric such that each cell division yields two distinct daughter cells, which differ with respect to their morphological and reproductive fates [13]. While one daughter, the stalked cell, initiates DNA replication immediately after cell division, the other daughter, the motile swarmer cell, is arrested in G1-phase and cannot initiate until after differentiation into a stalked cell. The replicative asymmetry of *Caulobacter* daughter cells ultimately stems from the asymmetric activation of CtrA, a response regulator that directly binds to and silences the origin of replication in swarmer, but not stalked cells [14,15]. CtrA is not critical, however, for preventing the re-initiation of DNA replication before cell division; like *E*. *coli*, and most other bacteria, the periodicity of replication initiation is dictated primarily by DnaA [16–18]. Similar to *E*. *coli*, a major mechanism controlling DnaA activity in *C*. *crescentus* is the stimulation of ATP hydrolysis upon initiation [17,19].

In contrast to *E*. *coli* and *Bacillus subtilis*, both of which possess multi-fork replication under fast-growth conditions [20,21], *Caulobacter* daughter cells are both born with one chromosome that replicates once-and-only-once per cell cycle [22]. Hence, *C*. *crescentus* does not require mechanisms to trigger multi-fork replication upon shift to nutrient-rich conditions. Nevertheless, *Caulobacter* must control the timing of replication initiation and cell division in response to nutritional changes or stress conditions to maintain genomic integrity. Prior studies have shown that the abundance of DnaA decreases rapidly following glucose starvation and on entry into stationary phase [23,24], although the mechanisms responsible are unclear. One study suggested that DnaA proteolysis is stimulated by glucose starvation [23], with a subsequent study demonstrating that the small signaling molecule (p)ppGpp is somehow involved in regulating DnaA stability following nutrient limitation [24]. In contrast to DnaA, CtrA is maintained upon carbon starvation and it was shown that (p)ppGpp and inorganic polyphosphate (polyP), another signalling molecule, are required for CtrA stability [25].

Although the mechanism(s) regulating DnaA during stationary phase and following carbon starvation remain unclear, recent work has provided insight into how DnaA abundance is adjusted following perturbations to the global state of cellular protein folding [26]. This work showed that the Lon protease degrades DnaA in *Caulobacter in vivo* and *in vitro* [26]. Degradation of DnaA by Lon occurs even in optimal growth conditions, but is stimulated even more upon the depletion of the DnaK chaperone or thermal stress, when unfolded proteins accumulate and the heat shock response is induced. Lon synthesis is upregulated as part of the heat shock response and, in addition, unfolded proteins appear to directly stimulate Lon to degrade DnaA [26]. Thus, the induction and stimulation of Lon blocks DNA replication initiation in proteotoxic stress conditions. In *E*. *coli* the activity of Lon in degrading ribosomal proteins and the antitoxins of toxin-antitoxin systems is stimulated by (p)ppGpp and polyP [27,28]. Whether Lon is required to modulate DnaA abundance in *Caulobacter* during nutrient starvation or stationary phase, and whether (p)ppGpp or polyP affect this degradation, remain unexplored.

Here, we investigated the mechanisms that drive a decrease in DnaA and DNA replication upon entry to stationary phase and following glucose exhaustion in *C*. *crescentus*. Our data demonstrate that Lon-mediated degradation is required in both conditions, but that the rate of proteolysis does not change significantly. Instead, we show that DnaA translation decreases as nutrients become scarce; this decrease in synthesis, combined with constitutive degradation by Lon rapidly eliminates DnaA and prevents DNA replication initiation. This mechanism depends on the 5´-untranslated leader region of the *dnaA* transcript, but does not depend on (p)ppGpp, indicating that another signal produced by nutrient limitation ultimately controls DnaA synthesis.

## Results

### Lon-dependent proteolysis is required to eliminate DnaA on entry to stationary phase

**Fig 1:**
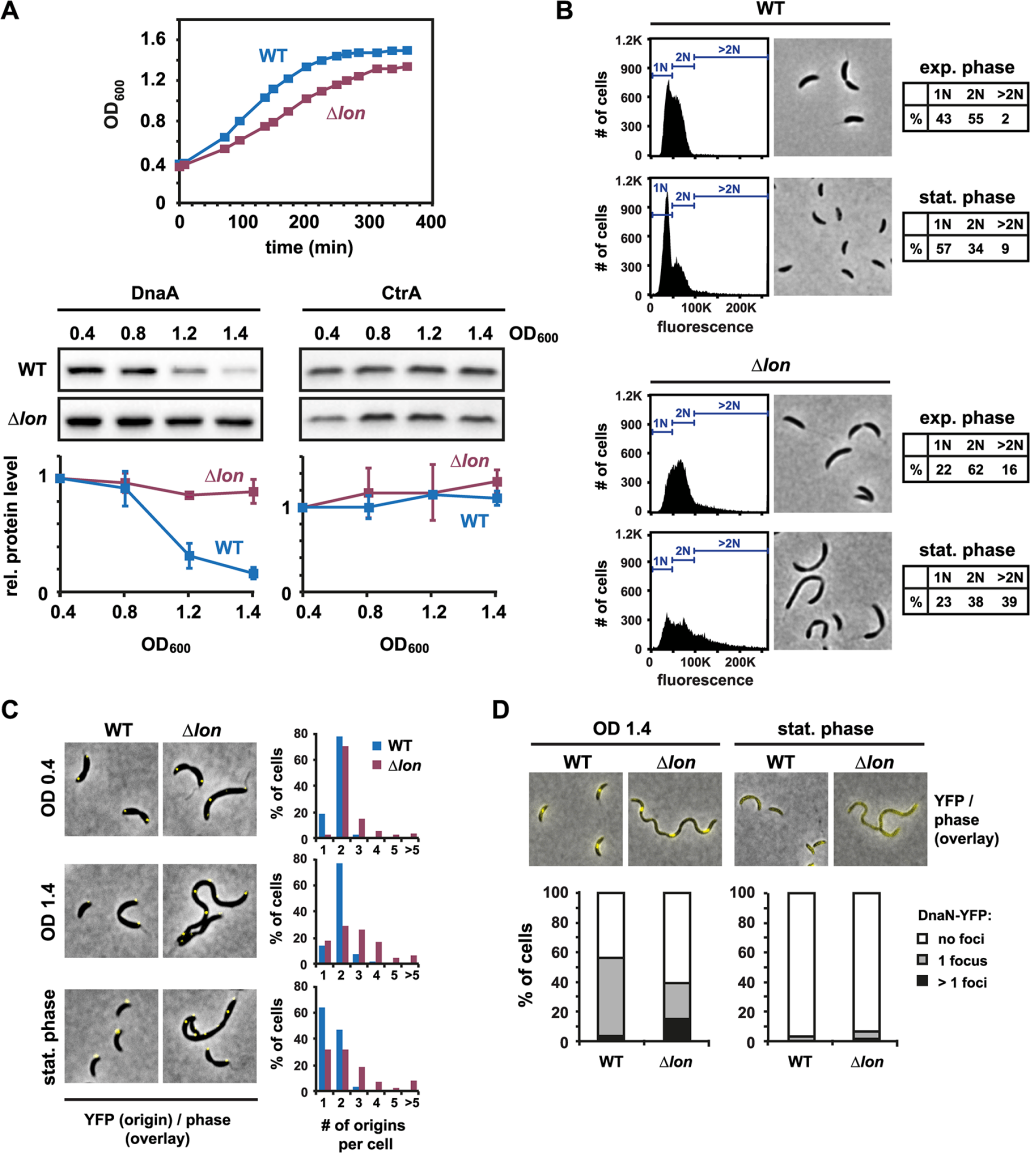
Lon-dependent proteolysis is required to eliminate DnaA and to induce a G1-arrest upon entry to stationary phase. (A) Growth phase-dependent changes in DnaA and CtrA protein levels in wild type (WT) and *Δlon* cells. The upper graphs show growth curves of WT and *Δlon* cells grown in rich medium (PYE). Western Blots show DnaA or CtrA protein levels at the indicated OD_600_ and after overnight growth in stationary phase (ON). The same set of samples was used in both Western blots. The bottom graphs show quantifications of band intensities. Averages of at least two independent replicates are shown with standard deviations. (B) Flow cytometry profiles and phase contrast microscopy images of wild type (WT) and *Δlon* cells in exponential phase (OD_600_ 0.4) or after growth for 24 hours in stationary phase. The percentage of cells containing one chromosome (1N), two chromosomes (2N) or more than two chromosomes (>2N) are shown in tables. (C) Number and subcellular localization of origins of replication in wild type and *Δlon* cells at OD_600_ 0.4, OD_600_ 1.4 and after growth for 24 hours at the maximum OD_600_ (stationary phase). Origins were labeled using a strain, which contains a *tetO* operator array close to the origin and the repressor gene *tetR-YFP* under the control of an inducible promoter. The number of origins per cell was quantified and graphically displayed. (D) DnaN-YFP foci in wild type and *Δlon* cells at OD_600_ 1.4 and after growth for 24 hours at the maximum OD_600_ (stationary phase). *dnaN-YFP* expression was induced by addition of 40 mM vanillate to the growth medium 1.5–2 hours prior to sampling. The number of foci was counted and graphically displayed.

The Lon protease degrades DnaA in *C*. *crescentus* and ensures a G1-arrest in conditions that lead to proteotoxic stress [26]. To investigate whether Lon also eliminates DnaA on entry to stationary phase, we first assessed changes in the steady-state levels of DnaA during the transition from exponential to stationary phase. We took samples from a culture of wild-type cells grown in rich medium to optical densities (OD_600_) of 0.4, 0.8, 1.2 and 1.4, and then measured DnaA levels by semi-quantitative Western blotting. DnaA protein abundance decreased as the culture reached higher optical densities, with the biggest decrease occurring between OD_600_ 0.8 and 1.2, when growth had slowed but not fully arrested (Fig 1A). In contrast to DnaA, the abundance of the response regulator CtrA, a negative regulator of DNA replication, remained relatively constant. Consistent with a reduction of DnaA and concomitant maintenance of CtrA, flow cytometry analysis indicated that most stationary phase cells (57%) contained a single chromosome indicating that cells were able to complete on-going rounds of DNA replication and the cell cycle but were blocked for initiating a new round of DNA replication (Fig 1B). Note that cells were analyzed 24 hours after reaching the maximal OD_600_ of 1.5 when cell size and morphology are similar to exponential phase cells (Fig 1B); prolonged periods in stationary phase of seven days result in the formation of elongated helical cells [29].

We also measured steady-state levels of DnaA at increasing cell densities in a strain containing a deletion of *lon* (Δ*lon*). The growth-phase dependent downregulation of DnaA was largely abolished in this mutant (Fig 1A); CtrA was not significantly affected. Flow cytometry analysis indicated that the majority of Δ*lon* cells (77%) were also not able to arrest the cell cycle with a single chromosome. Instead, a considerable number of Δ*lon* cells (39%) grown in stationary phase contained more than two chromosomes and were somewhat filamentous (Fig 1B). Consistent with these flow cytometry data, using the fluorescence repressor-operator system (FROS), which fluorescently marks origins of replication [30], we observed that Δ*lon* cells grown to stationary phase often contained two or more origins per cell (Fig 1C). By contrast, most wild type cells in stationary phase have only one origin. To investigate whether DNA replication is ongoing in the Δ*lon* mutant in stationary phase, we monitored the localization of the replisome by expressing an ectopic copy of *dnaN-YFP*. Wild-type cells rarely habor more than one DnaN-YFP focus (Fig 1D). By contrast, a significant number of Δ*lon* cells contained more than one DnaN-YFP foci at OD_600_ 1.4, indicating that in these cells multiple replisomes replicate the DNA. Notably, after 24 hours of growth in stationary phase, in both wild type and Δ*lon* cells DnaN-YFP foci were no longer detectable, suggesting that at this time point DNA replication no longer takes place and that cells arrest with the number of chromosomes that they accumulated early on in stationary phase.

Altogether, these data demonstrate that the Lon protease is required to eliminate DnaA at the entry to stationary phase and to ensure that cells contain a single fully replicated chromosome when entering stationary phase.

### (p)ppGpp is not required to eliminate DnaA during the entry to stationary phase

**Fig 2:**
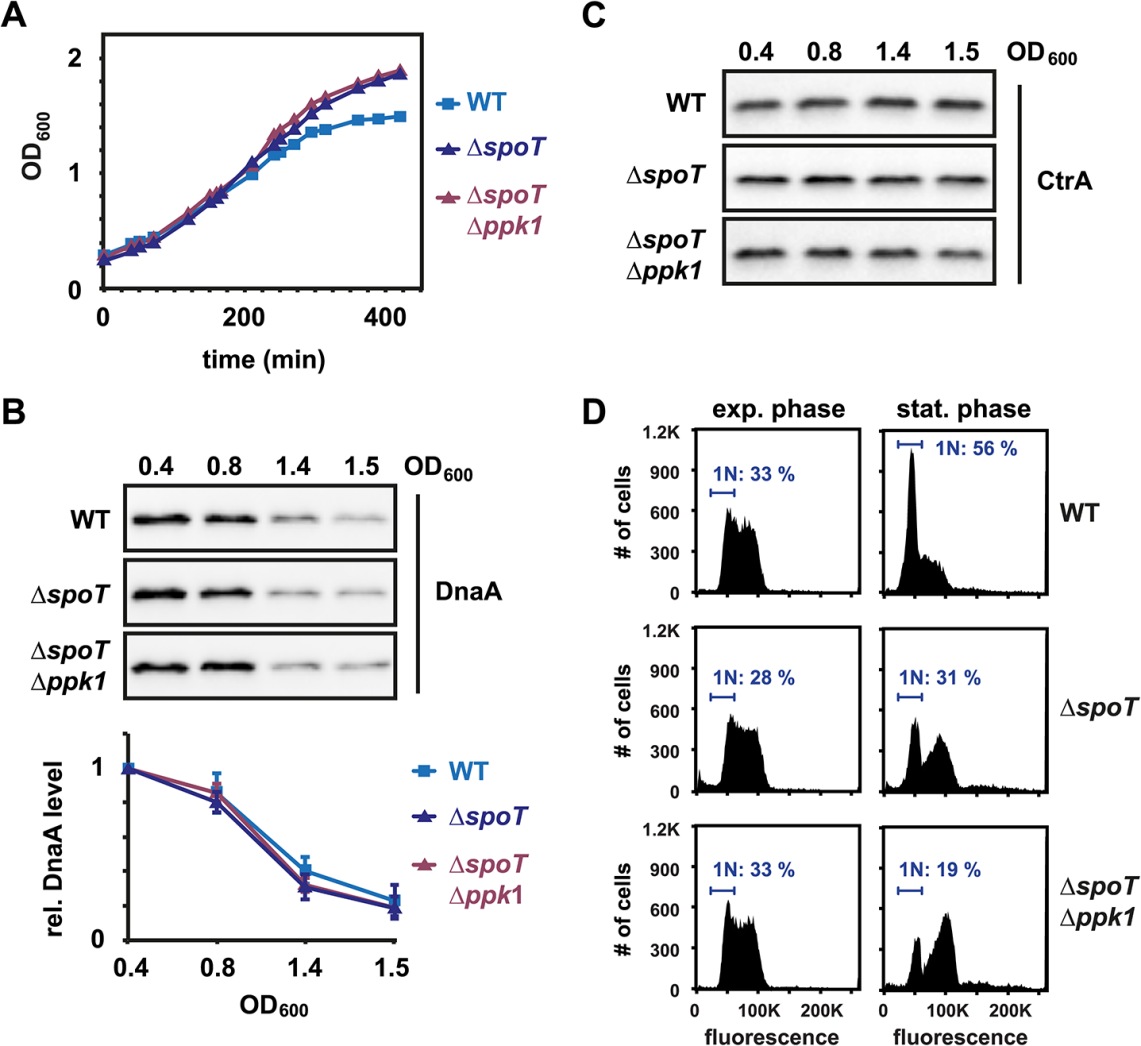
(p)ppGpp is not required to eliminate DnaA during the entry to stationary phase. (A) Growth curves of wild type (WT), *ΔspoT* and *ΔspoTΔppk1* cells grown in PYE. (B) Western Blots showing DnaA protein levels at the indicated optical densities in the three strains. The graphs show quantifications of band intensities. Averages of at least two independent replicates are shown with standard deviations. See also S1 Fig for DnaA stability in *ΔspoT* cells. (C) Western Blots as in (B), but probed with an antibody specific for CtrA. (D) Flow cytometry profiles of WT, *ΔspoT* and *ΔspoTΔppk1* cells in exponential phase (OD_600_ 0.4) or after growth for 24 hours in stationary phase. The percentage of cells with one chromosome (1N) is indicated.

(p)ppGpp was previously suggested to affect DnaA accumulation in *C*. *crescentus* and *E*. *coli* [24,25,31,32]. Furthermore, in *E*. *coli* (p)ppGpp and polyP can trigger Lon to degrade ribosomal proteins and antitoxins [27,28]. To analyze the contribution of (p)ppGpp and polyP to the regulation of DnaA abundance and DNA replication during entry to stationary phase, we constructed strains containing a deletion of *spoT*, which encodes the only (p)ppGpp synthase in *C*. *crescentus* [24], or both a *spoT* deletion and a deletion of *ppk1*, which encodes a polyphosphate kinase that drives polyP synthesis [25,33,34]. Both strains grew to a higher OD_600_ (~2.0) than the wild type (OD_600_ ~ 1.5) (Fig 2A), supporting earlier reports that an inability to produce (p)ppGpp promotes a proliferative mode [25,35]. To test if (p)ppGpp is responsible for the downregulation of DnaA in stationary phase, we compared DnaA levels in the Δ*spoT* and Δ*spoT*/Δ*ppk1* mutants with the wild type. DnaA was eliminated from the mutant cells in a similar manner as in wild-type cells during entry to stationary phase (Fig 2B) and *in vivo* degradation assays showed that the stability of DnaA was nearly identical in wild-type and Δ*spoT* cells (S1 Fig). The levels of CtrA were also not strongly affected by the deletion of *spoT* and *ppk1* in the conditions tested, suggesting that (p)ppGpp does not play a major role in adjusting DnaA and CtrA levels during the entry to stationary phase (Fig 2C). Consistent with the clearing of DnaA and maintenance of CtrA, flow cytometry analysis indicated that cells lacking *spoT* arrested DNA replication initiation, although many cells arrested with two chromosomes rather than one (Fig 2D). This may indicate a disruption of a later cell cycle step in *spoT* mutants during the entry to stationary phase.

### Downregulation of DnaA on entry to stationary phase is not due to faster proteolysis

**Fig 3:**
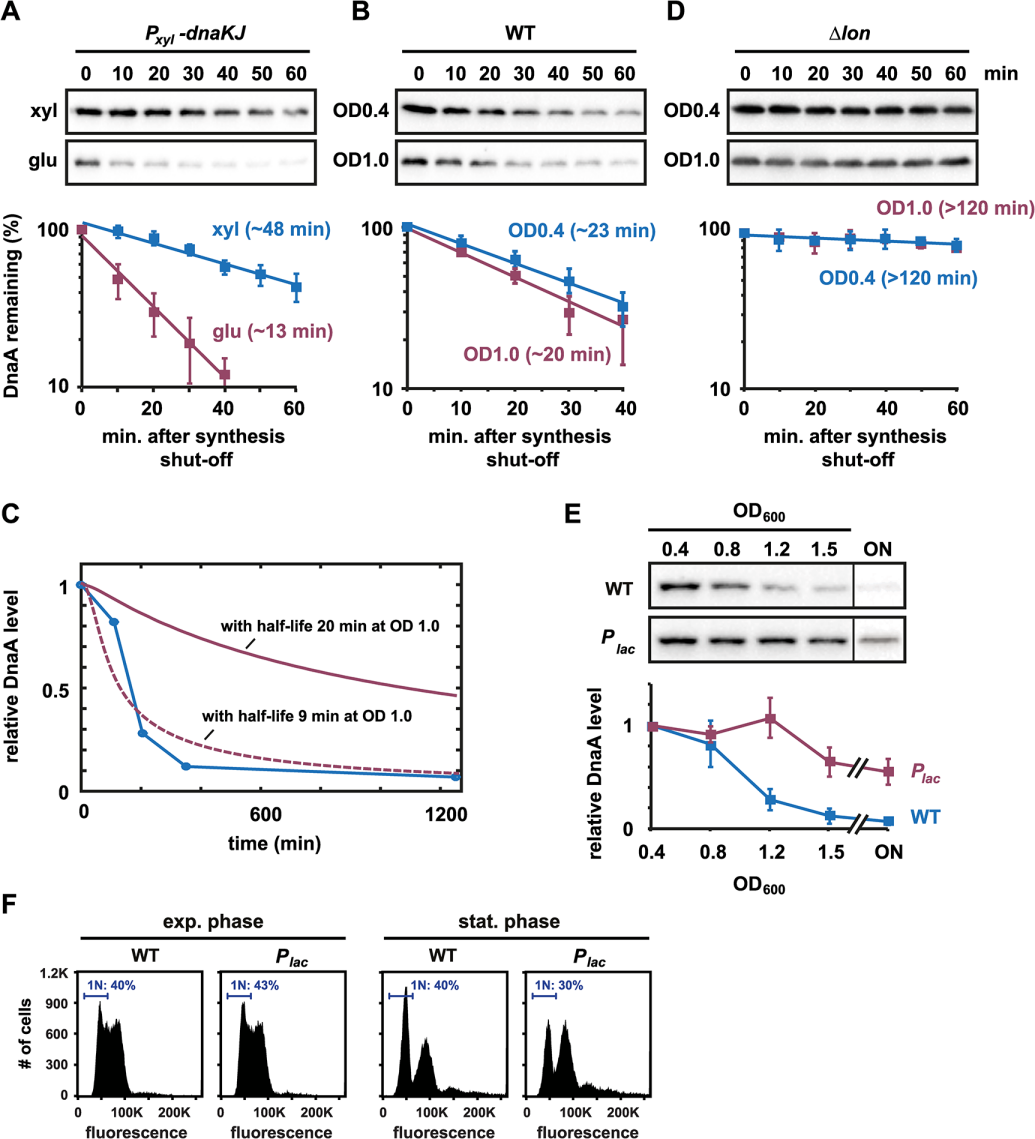
Downregulating DnaA on entry to stationary phase is not due to faster proteolysis, but changes in DnaA synthesis. (A) *In vivo* degradation assays showing DnaA stability in the DnaK/J depletion strain. Cells were grown in PYE with xylose (blue) or PYE with glucose for 4.5 hrs (red) to deplete DnaK/J, before chloramphenicol was added to shut-off protein synthesis. DnaA abundance was monitored by Western Blotting. Band intensities were quantified (bottom); averages of two independent replicates are shown with standard deviations (see also S2 Fig). (B) *In vivo* degradation assays showing DnaA stability in wild type cells during exponential growth (OD_**600**_ 0.4) and at the onset of stationary phase (OD_**600**_ 1.0). Band intensities of Western Blots (top) were quantified (bottom); averages of three independent replicates are shown with standard deviations (see also S2 Fig). (C) Modeled protein abundance over the growth curve for different DnaA protein half-lives. The blue line shows measured changes in DnaA abundance over time as cells are grown to stationary phase (see also Figs 1A and 3E). The solid red line shows predicted changes in protein abundance, assuming that degradation increases in a linear manner, protein synthesis is constant and that the half-life of DnaA is 23 min at OD_**600**_ 0.4 and 20 min at OD_**600**_ 1.0 (see Materials and Methods for the mathematical model). The dashed red line shows predicted protein abundance if the half-life is 23 min at OD_**600**_ 0.4 and 9.1 min at OD_**600**_ 1.0, the latter value having been found by best fit to the data. (D) *In vivo* degradation assays showing DnaA stability in *Δlon* cells during exponential growth (OD_**600**_ 0.4) or at the entry into stationary phase (OD_**600**_ 1.0). Band intensities of Western Blots (top) were quantified (bottom); averages of two independent replicates are shown with standard deviations (see also S2 Fig). (E) Growth phase-dependent changes in DnaA protein levels in wild type and a strain in which the native copy of *dnaA* is under the control of an IPTG inducible promoter (*P*_***lac***_). 1 mM IPTG was added to the culture to induce constitutive *dnaA* expression (see also S4 and S5 Figs). The bottom graphs show the average band intensity of at least three independent experiments with standard deviations. (F) Flow cytometry profiles of wild type (WT) and the *P*_***lac***_ ***-*** *dnaA* strain in exponential phase (OD_**600**_ 0.4) or after growth for 24 hours in stationary phase. The percentage of cells with one chromosome (1N) is indicated.

A reduction in DnaA levels during the entry to stationary phase may result from increased proteolysis or decreased synthesis, or both. Previous work showed that proteotoxic stress, resulting from chaperone depletion or acute heat shock, can increase the rate of DnaA proteolysis [26]. To investigate whether the rate of DnaA degradation is affected by growth phase, we measured DnaA stability *in vivo* by adding chloramphenicol to cells to stop protein synthesis and then assessed DnaA decay rates over time by immunoblotting. As documented previously [26], DnaA stability decreases significantly, from ~48 min. to ~13 min., in cells depleted of the chaperone DnaK due to increased degradation by Lon (Figs 3A and S2). In contrast, the half-life of DnaA at OD_600_ ~ 1.0 was only slightly shorter (20 min.) than at OD_600_ ~ 0.4 (23 min.) (Figs 3B and S2). Using a simple model for DnaA abundance (see Methods), we determined that a difference in protein half-life of three minutes could produce at most a 25% decrease in DnaA abundance between an OD_600_ of 0.4 and an OD_600_ of 1.5, 400 min later (Fig 3C). To generate the observed 90% drop in DnaA abundance over the same OD_600_ range would require that the half-life decreases to ~9.1 min by OD_600_ ~ 1.0 (in comparison to the measured value of 20 min). We also measured DnaA stability at OD_600_ ~ 1.2 and did not detect a difference in half-life greater than three minutes when compared to OD_600_ ~ 0.4 (S3 Fig). Thus, we conclude that a change in protein half-life cannot explain the change in DnaA abundance that occurs at the onset of stationary phase.

Although growth phase had little effect on DnaA stability, deleting the Lon protease had a strong effect on DnaA stability. In Δ*lon* cells, DnaA had a half-life >120 min. in both exponential and early stationary phase cells (Figs 3D and S2), reinforcing previous results that DnaA degradation depends strongly on Lon [26]. The stabilization of DnaA in Δ*lon* cells agrees with the finding that Δ*lon* cells fail to timely eliminate DnaA at the entry to stationary phase (Fig 1A). Taken together, our data indicate that Lon is required to efficiently clear DnaA at the onset of stationary phase, but, importantly, that the rate of Lon-dependent degradation of DnaA is not substantially changed upon stationary phase entry.

### Constitutive expression of DnaA from a *P*_*lac*_ promoter prevents DnaA downregulation

Because regulated degradation does not explain the growth phase-dependent decrease in DnaA abundance, we thought that changes in DnaA levels likely stem from changes in DnaA synthesis. To test this possibility, we used a strain in which the promoter of *dnaA* and its 5´ untranslated leader region (5'UTR) were replaced by *P*_*lac*_, an IPTG-regulated promoter, and its native leader. Addition of 1 mM IPTG to the growth medium resulted in constitutive *dnaA* expression with DnaA levels comparable to those seen in wild-type cells grown to exponential phase in rich medium (S4 Fig). We then followed DnaA abundance in this strain from exponential phase into stationary phase. In contrast to the wild type, the *P*_*lac*_ *-dnaA* strain was unable to clear DnaA upon entry to stationary phase, with DnaA levels remaining relatively constant up to an OD_600_ of 1.2 (Figs 3E and S5). DnaA levels dropped by ~40% once cells were at OD_600_ ~ 1.5, although DnaA levels were decreased by nearly 90% at the same density in wild-type cells. These data demonstrate that constitutive expression of *dnaA* is sufficient to bypass the downregulation of DnaA at high cell density, in agreement with our finding that DnaA degradation is not significantly changed upon entry to stationary phase (Fig 3B). Moreover, flow cytometry analysis demonstrated that the number of cells harboring a single chromosome in stationary phase was reduced in the *P*_*lac*_-*dnaA* strain compared to wild type (Fig 3F). Notably, however, *P*_*lac*_-*dnaA* cells did not accumulate extra chromosomes as seen with Δ*lon* cells grown to stationary phase (Fig 1B), suggesting that the Δ*lon* phenotype likely results from an increased stability of DnaA and other Lon substrates.

### Reduced translation of *dnaA* accounts for the downregulation of DnaA abundance at the onset of stationary phase

**Fig 4:**
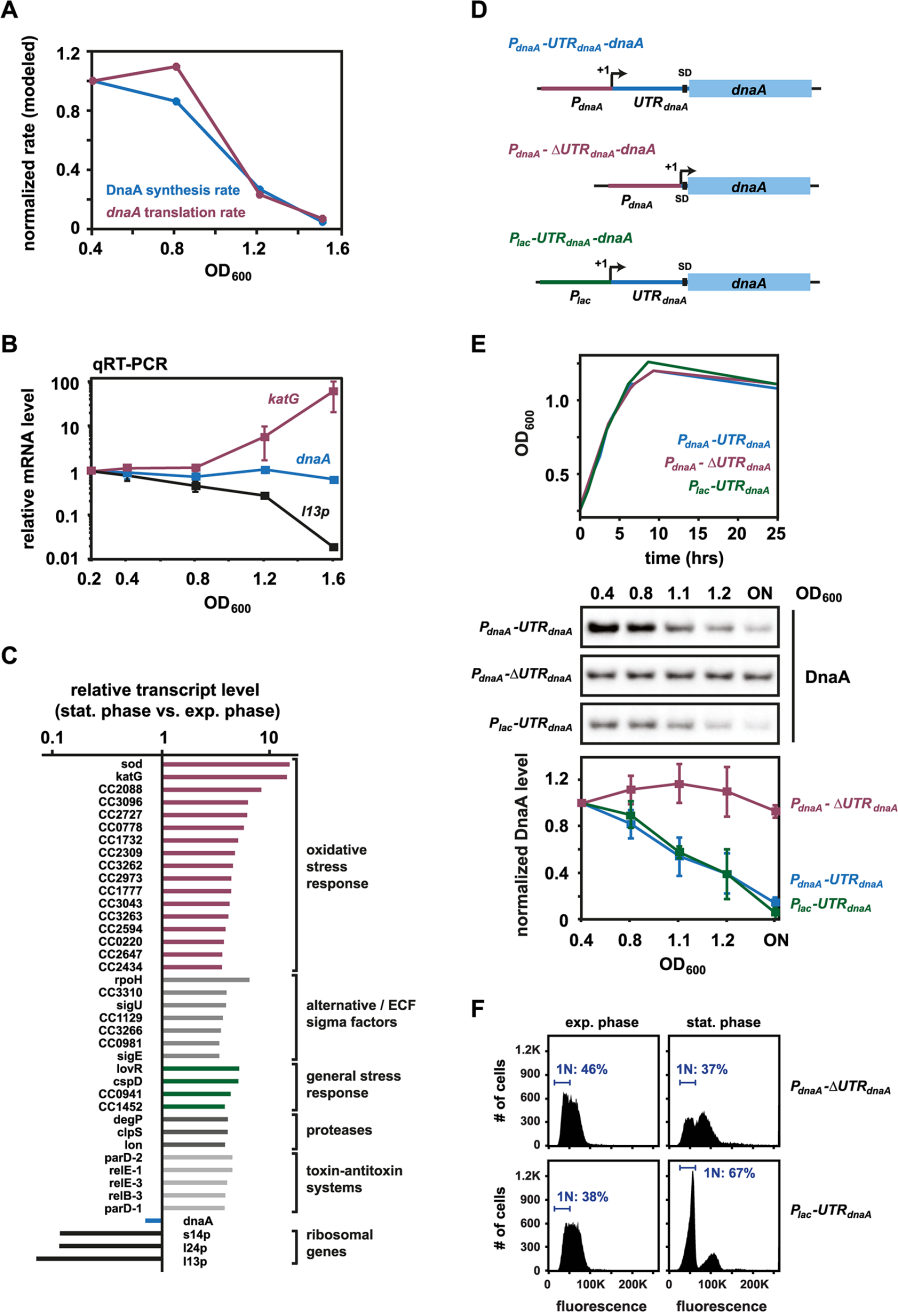
Reduced translation of *dnaA* accounts for the downregulation of DnaA abundance at the onset of stationary phase. (A) Modeled DnaA synthesis (blue) and *dnaA* translation (red) rates over the growth curve in wild type *C*. *crescentus*. Synthesis and translation rates were mathematically determined as described in the Materials and Methods. (B) Transcript levels of *dnaA*, *katG* and *l13p* at the indicated optical densities in a wild type culture as determined by qPCR. Average values of relative expression changes of two independent experiments are shown with standard deviations. (C) Transcript levels as determined by microarray analysis of *dnaA*, *katG* and *l13p* as well as selected genes involved in stress responses in wild type grown to late stationary phase. Levels are relative to transcript levels of a culture grown in exponential phase. (D) Schematics of different expression constructs (not to scale), which either contain or lack the 5'UTR of the *dnaA* gene. The constructs were expressed from a low copy plasmid in a strain background in which the native copy of *dnaA* is under the control of a xylose inducible promoter (strain GM2471). (E) Changes in DnaA protein over the growth curve in strains expressing either of the three constructs shown in (D). The bottom graphs show the average band intensity of at least two independent experiments with standard deviations. All strains were grown in the absence of xylose to shut off *dnaA* expression from the chromosome. The strain harboring the construct *P*_***lac***_ *-UTR*_***dnaA***_ *-dnaA* was grown in the presence of 1 mM IPTG (see also S6 Fig). (F) Flow cytometry profiles of the strains carrying plasmids that either lack (*P*_***dnaA***_ *-ΔUTR*_***dnaA***_ *-dnaA*) or contain (*P*_***lac***_ *-UTR*_***dnaA***_ *-dnaA*) the 5'UTR of the *dnaA* gene. Cells were grown for 12 hours after reaching the maximal OD_**600**_ before samples were taken for flow cytometry analysis. The percentage of cells with one chromosome (1N) is indicated.

Our results with P_*lac*_-*dnaA* strongly suggest that changes in DnaA synthesis cause DnaA levels to drop during entry to stationary phase. Knowing DnaA steady-state levels, DnaA half-life, and cell growth rates at different optical densities allowed us to infer how the rate of DnaA synthesis changes as a function of culture density using a mathematical model. Our modeling predicted that DnaA synthesis drops approximately 20-fold between OD_600_ 0.4 and OD_600_ 1.5 (Fig 4A). To test if this change in DnaA synthesis results from changes in *dnaA* transcription or mRNA stability, we measured *dnaA* mRNA levels using quantitative real-time RT-PCR (qPCR) on samples from a culture grown to an OD_600_ of 0.2, 0.4, 0.8, 1.2, and 1.6. Unexpectedly, *dnaA* mRNA levels did not vary significantly as a function of culture density (Fig 4B). Even at an OD_600_ of 1.6, *dnaA* mRNA levels did not fall below 65% of transcript levels measured in exponential phase. By contrast, *katG*, a known stationary phase-induced gene [36] was upregulated more than 60-fold at OD_600_ 1.6, and *l13p*, encoding a ribosomal protein that is repressed during stationary phase, was downregulated more than 50-fold (Fig 4B). Consistent with our qPCR results, DNA microarray analysis showed that *dnaA* transcript levels were not substantially changed in stationary phase (70% of exponential phase levels), again in contrast to *katG* and *l13p*, which showed significant induction and repression, respectively (Fig 4C). These results show that although the rate of DnaA synthesis strongly declines at the onset of stationary phase, *dnaA* mRNA abundance does not, implying that DnaA translation is likely the growth-phase regulated step in DnaA synthesis. Incorporating the qPCR results into our mathematical model, we inferred that the rate of *dnaA* translation during the transition to stationary phase must decline to approximately 5% of the rate during exponential phase growth (Fig 4A).

Changes in translation often involve the 5' untranslated region, or leader, of bacterial mRNAs. In *C*. *crescentus*, *dnaA* contains a relatively long 5' leader of 155 nt [37,38], which was previously shown to affect *dnaA* expression during exponential phase [39]. To test if the 5' leader also plays a role in modulating DnaA synthesis at the onset of stationary phase, we placed the coding region of *dnaA* under the control of the native *dnaA* promoter, but without 140 nt of the leader, retaining only the region of the leader containing the native Shine-Dalgarno sequence (Fig 4D). This construct was cloned into a low-copy vector and transformed into a strain in which the chromosomal copy of *dnaA* could be depleted by growing cells in the absence of xylose. As a control, we used a plasmid in which *dnaA* is controlled by the entire upstream region of the native *dnaA* locus, including the promoter and the 5' UTR (Fig 4D). With the control plasmid, DnaA was eliminated when the culture reached high optical density as growth rate starts to decline, as in wild-type cells (Figs 1A and 4E). Strikingly however, with the plasmid lacking the 5' leader, DnaA was no longer downregulated upon entry to stationary phase (Figs 4E and S6), demonstrating that the 5' leader is required for the growth-phase-dependent decrease in DnaA abundance. In contrast to wild type cells, which arrest the cell cycle with a single chromosome in stationary phase (Fig 1B), the strain carrying the plasmid lacking the 5'UTR was not able to arrest in G1-phase (Fig 4F). To test if the 5' leader of *dnaA* is sufficient to induce a downregulation of protein abundance at the entry to stationary phase, we placed *dnaA*, with its 5' leader, under control of a *P*_*lac*_ promoter (Fig 4D). A strain harboring this construct grown in the presence of IPTG showed a significant downregulation of DnaA upon entry into stationary phase, similar to that seen in wild type cells (Figs 4E and S6). This downregulation of DnaA was sufficient to allow cells to arrest the cell cycle with a single chromosome in G1-phase in stationary phase (Fig 4F).

In sum, our data suggest that as cells transition from exponential to stationary phase, translation of the *dnaA* mRNA decreases significantly; because DnaA has a relatively short half-life, due to constitutive degradation by Lon, this drop in translation leads to a relatively rapid decrease in the abundance of the replication initiator and a consequent G1-arrest.

### The translation of *dnaA* is modulated by nutrient availability

**Fig 5:**
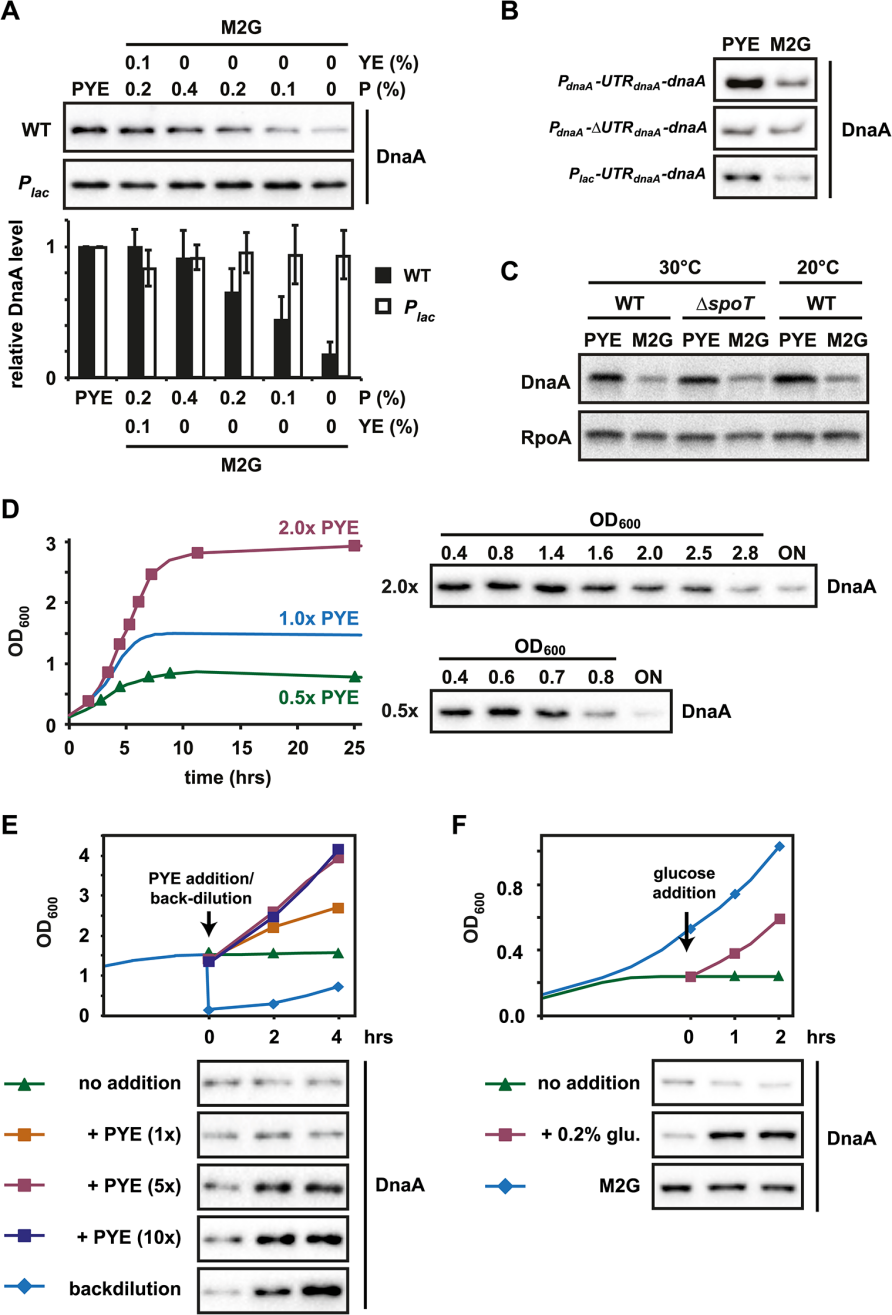
The translation of *dnaA* is modulated by nutrient availability. (A) DnaA protein levels in exponentially grown wild type or *P*_*lac*_ cells when cultured in PYE or in M2G (minimal medium) supplemented with the indicated amounts of peptone (P) or peptone and yeast extract (YE). Band intensities were quantified (bottom); data points represent averages of two independent experiments with standard deviations (see also S3 Fig). (B) Growth medium-dependent DnaA protein abundance in strains expressing constructs, which either contain or lack the 5'UTR of *dnaA* (see Fig 4D). The three strains were grown in PYE and M2G and protein abundance was measured by Western blotting. The strain harboring the construct *P*_*lac*_ *-UTR*_*dnaA*_ *-dnaA* was grown in the presence of 1 mM IPTG (see also S7 Fig). (C) Protein levels of DnaA and RpoA (loading control) in wild type and *ΔspoT* cells in PYE and M2G when grown at 30°C and in wild type cells when grown in PYE and M2G at room temperature (20°C). (D) Growth phase-dependent changes in DnaA protein levels in wild type cells when grown in 2x (red) and 0.5x (green) PYE. Cells were grown in the respective media to stationary phase. DnaA protein abundance was measured at the indicated culture OD_600_ by Western blotting (see also S7 Fig). The growth curve for wild type grown in 1x PYE is shown for comparison and is reproduced from Fig 1A. (E) Changes in growth rate (upper graph) and DnaA protein levels after nutrient addition to a culture grown in stationary phase. A culture was grown for two hours in stationary phase (at OD_600_ 1.5) before concentrated PYE was added to a final concentration of 1x (orange), 5x (red) or 10x (dark blue) that of PYE medium. As controls, the culture was either maintained in stationary phase (no addition, green) or backdiluted (1:10) into fresh PYE medium (backdilution, blue). DnaA protein levels were analyzed by Western blotting at the indicated time points (see also S7 and S8 Figs). (F) Changes in growth rate (upper graph) and DnaA after glucose addition to a carbon starved culture. A culture grown in M2G was shifted to M2 medium containing 0.02% glucose to induce carbon starvation. Two hours after the resulting growth arrest the culture was split into two subcultures. One of them remained untreated (no addition, green line), the other culture was supplemented with 0.2% glucose (glucose addition, red line). A third culture was grown in M2G medium throughout the experiment (M2G, blue line). DnaA protein levels were analyzed by Western blotting at the indicated time points (see also S7 and S8 Figs).

A reduction in growth rate during the entry into stationary phase may result from the exhaustion of nutrients or the accumulation of inhibitory waste products, cellular stress, or some combination thereof [40]. We hypothesized that a decrease in nutrient availability might be the signal that ultimately modulates *dnaA* translation. To test this idea we analyzed DnaA accumulation in growth media containing different amounts of nutrients. M2G, a minimal medium, in which the sole carbon source is glucose, was used as the most nutrient poor medium. We supplemented M2G with increasing amounts of peptone, a pepsin digest consisting of polypeptides and amino acids used in rich media such as PYE. DnaA protein levels during mid-exponential phase were clearly correlated with the complexity of the growth medium (Fig 5A). Increases in the amount of peptone added to M2G were mirrored by increases in DnaA steady-state levels, as measured by Western blotting. In contrast to DnaA, levels of the Lon protease were relatively unaffected by the growth medium (S7 Fig).

We performed the same experiment using a *P*_*lac*_ *-dnaA* strain in which *dnaA* lacks its native promoter and 5' leader. Growing this strain in the presence of 1 mM IPTG caused DnaA levels to be relatively constant and independent of the growth medium (Fig 5A), demonstrating that nutrient-dependent changes in DnaA protein levels likely depend on changes in DnaA synthesis, not proteolysis. Moreover, in a strain containing the construct *P*_*dnaA*_ *-ΔUTR*_*dnaA*_ *-dnaA* (Fig 4D) in which *dnaA* is regulated by its native promoter but lacks its usual long leader sequence, DnaA levels did not differ between M2G and PYE (Figs 5B and S7), strongly suggesting that the 5'UTR leader of DnaA is responsible for nutrient-dependent changes in protein levels. Consistent with this conclusion, we found that the construct *P*_*lac*_ *-UTR*_*dnaA*_ *-dnaA* showed a growth-medium-dependent accumulation of DnaA, similar to wild type (Figs 5B and S7). Neither a deletion of *spoT* nor a lower temperature, which decreases growth rate, had a significant effect on DnaA abundance in the two different media (Fig 5C).

To further examine the correlation between nutrient availability, growth rate and changes in DnaA abundance, we grew *C*. *crescentus* in PYE medium, which contained either higher (2x PYE) or lower (0.5x PYE) amounts of nutrients, respectively, and followed DnaA abundance along the growth curve. In 2x PYE medium cultures reached stationary phase at OD_600_ 2.8; by contrast, growth in 0.5x PYE led to a growth arrest at OD_600_ 0.8 (Fig 5D). DnaA levels dropped in both conditions concomitantly with the cessation of growth, consistent with the hypothesis that changes in DnaA abundance coincide with nutrient exhaustion and a slowdown of the growth rate (Figs 5D and S7).

Next, we wanted to test if cells that have already reached stationary phase can accumulate DnaA after adding nutrients to the culture. To address this question we added concentrated nutrients (final concentration 1x, 5x or 10x of nutrients in PYE medium) to a culture grown for two hours in stationary phase at OD_600_ ~ 1.5, and then monitored subsequent changes in growth rate, DnaA levels and DNA replication. Addition of 1x PYE led only to a slow increase in growth rate, likely because the fresh nutrients are quickly consumed by the high-density culture leading to a re-entry into stationary phase (Figs 5E and S7). In this condition DnaA levels remained relatively low. By contrast however, addition of 5x or 10x PYE nutrients allowed cells to resume rapid growth, which was nearly as fast as the growth of a culture that was backdiluted from stationary phase into fresh PYE medium. In these conditions DnaA levels increased within two hours and flow cytometry analysis showed that cells were able to initiate DNA replication (Figs 5E and S8). These data show that cells that have reached a high OD are able to upregulate DnaA and initiate DNA replication when sufficient amounts of fresh nutrients are added to allow for rapid growth. Altogether these results reinforce our model that DnaA levels and DNA replication are tightly linked to nutrient availability and cellular growth rate.

### Elimination of DnaA upon carbon exhaustion depends on Lon and regulated DnaA synthesis, but not on (p)ppGpp

**Fig 6:**
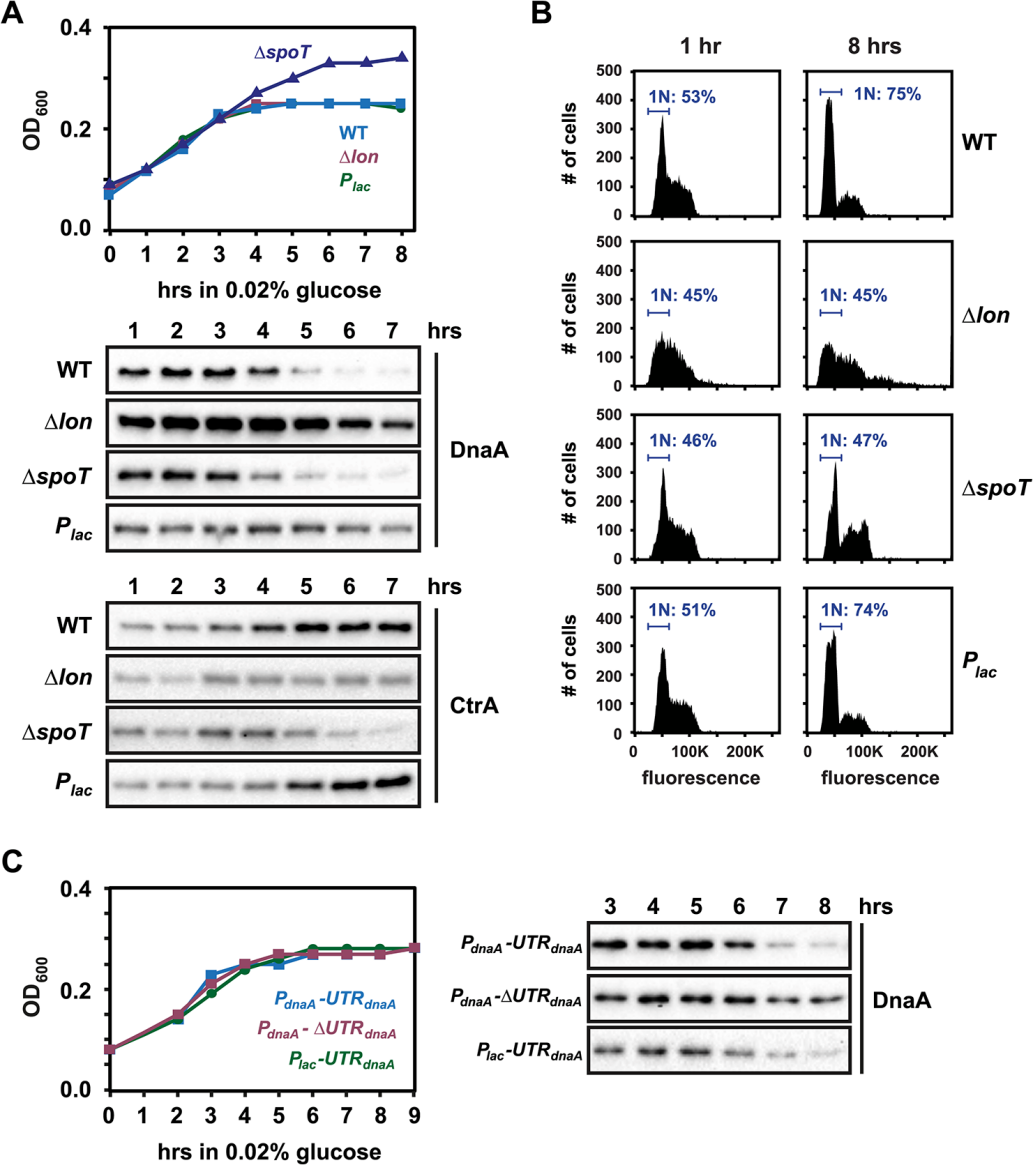
Regulated DnaA synthesis and Lon-mediated degradation are required to eliminate DnaA upon carbon exhaustion. (A) Growth curves and Western Blots showing changes in DnaA and CtrA levels after shifting wild type, *Δlon*, *ΔspoT* and *P*_*lac*_ *-dnaA* cells from M2G to M2 medium containing 0.02% glucose at t = 0. The culture of *P*_*lac*_ *-dnaA* cells was supplemented with 50 μM IPTG to induce *P*_*lac*_. See also S9 Fig for DnaA stability before and after glucose exhaustion. (B) Flow cytometry profiles of wild type, *Δlon*, *ΔspoT* and *P*_*lac*_ *-dnaA* cells 0 or 8 hours after shift from M2G to M2 medium containing 0.02% glucose. The percentage of cells with one chromosome (1N) is indicated. (C) Growth curves and Western Blots showing changes in DnaA levels in strains, which either contain or lack the 5'UTR (see Fig 4D), after shift from M2G to M2 medium containing 0.02% glucose at t = 0 (see also S10 Fig). All strains were grown in the absence of xylose to shut off *dnaA* expression from the chromosome. The strain harboring the construct *P*_*lac*_ *-UTR*_*dnaA*_ *-dnaA* was grown in the presence of 1 mM IPTG.

Previous work demonstrated that rapid carbon starvation can also lead to the elimination of DnaA [23–25]. To monitor DnaA levels in starvation conditions, we performed a glucose exhaustion assay in which cultures grown in M2G were shifted to M2G_1/10_, which contains 10% of the glucose in M2G. Initially, cells continued growing; however, as glucose in the medium was consumed, the density of the culture stopped increasing, leveling off at OD_600_ ~ 0.25, approximately 4 hours after the shift to M2G_1/10_ (Fig 6A) [25]. Concomitant with this growth arrest, DnaA levels dropped and cells arrested in G1-phase (Fig 6A and 6B). We measured the stability of DnaA in M2G and four hours after shifting cells to M2G_1/10_, the time point when growth arrested and DnaA abundance drfopped most strongly. However, we did not detect changes in DnaA stability (S9 Fig).

Cells harboring a Δ*lon* mutation contained approximately 2–3 fold higher DnaA levels and were not able to efficiently clear DnaA upon glucose exhaustion (Fig 6A). Likewise, constitutive expression of DnaA from the *P*_*lac*_ promoter abolished downregulation of DnaA during glucose exhaustion. Furthermore, by using the different plasmid-borne constructs that either contain or lack the 5´UTR of *dnaA* (Fig 4D), we found that the downregulation of DnaA upon carbon exhaustion strongly depended on the presence of this region of the *dnaA* mRNA (Figs 6C and S10). Together, these findings suggest that, as in stationary phase, the adjustment of DnaA abundance is mediated by the combined effects of regulated translation and constant proteolysis. In the Δ*spoT* mutant, DnaA was eliminated as in wild-type cells despite growth to a higher final OD_600_ (Fig 6A), similar to the situation in stationary phase.

Flow cytometry analysis showed that glucose starvation led to a G1-arrest in the wild type, but not in the Δ*lon* mutant (Fig 6B). Notably, the *P*_*lac*_ *-dnaA* strain still exhibited a G1-arrest in most cells despite the maintenance of DnaA levels, demonstrating that in this condition the availability of DnaA is not sufficient for DNA replication initiation and that another mechanism exists that blocks replication. One possibility is that DnaA is not active for DNA replication in this condition. Alternatively, CtrA binding to the origin might block DnaA's access to the origin [41]. Indeed, it has been shown previously that CtrA is stabilized in starved swarmer cells [23,25]. Consistent with this finding, we observed a significant increase in CtrA levels upon glucose exhaustion in wild type and *P*_*lac*_ *-dnaA* cells (Fig 6A). By contrast, CtrA was not upregulated in Δ*lon* and Δ*spoT* strains (Fig 6A). Together these data demonstrate that both DnaA and CtrA are tightly, and reciprocally, regulated to ensure that DNA replication does not initiate upon carbon starvation. In addition, the nucleotide bound state of DnaA might be affected under starvation conditions.

We tested if the addition of glucose to a carbon-starved culture can restore DnaA levels and DNA replication, performing a similar nutrient re-addition experiment as above (Fig 5E). In this case we shifted wild-type cells from M2G to M2G_1/10_ to deplete glucose. After growth had been arrested for two hours and DnaA was no longer detectable, we added glucose back to the culture at a final concentration of 0.2%. Growth of the culture quickly resumed, with a rate similar to a culture that was kept in M2G throughout the experiment (Fig 5F) and DnaA levels were rapidly upregulated after glucose addition (Figs 5F and S7). Moreover, the number of cells in S-phase quickly increased after glucose addition, indicating that cells started to initiate DNA replication (S8 Fig). Hence, in carbon-starved cells the lack of nutrients is the only reason for low DnaA protein levels; the addition of nutrients rapidly restores DnaA levels and DNA replication.

## Discussion

Nearly all bacteria depend on DnaA for chromosome replication and viability. In several bacteria, DnaA has also been shown to regulate transcription [42–44]. Due to these important functions, the activity and cellular concentration of DnaA must be tightly regulated. In *C*. *crescentus*, the abundance of DnaA is strongly downregulated under different stress conditions, providing an efficient way to block or delay DNA replication and cell cycle progression until conditions improve. Our previous work already revealed a mechanism of how DnaA is downregulated under proteotoxic stress conditions [26]. However, the mechanism ensuring DnaA downregulation under starvation conditions has, until now, been unclear.

### Dynamic control of DnaA abundance by regulated translation and constant degradation

**Fig 7:**
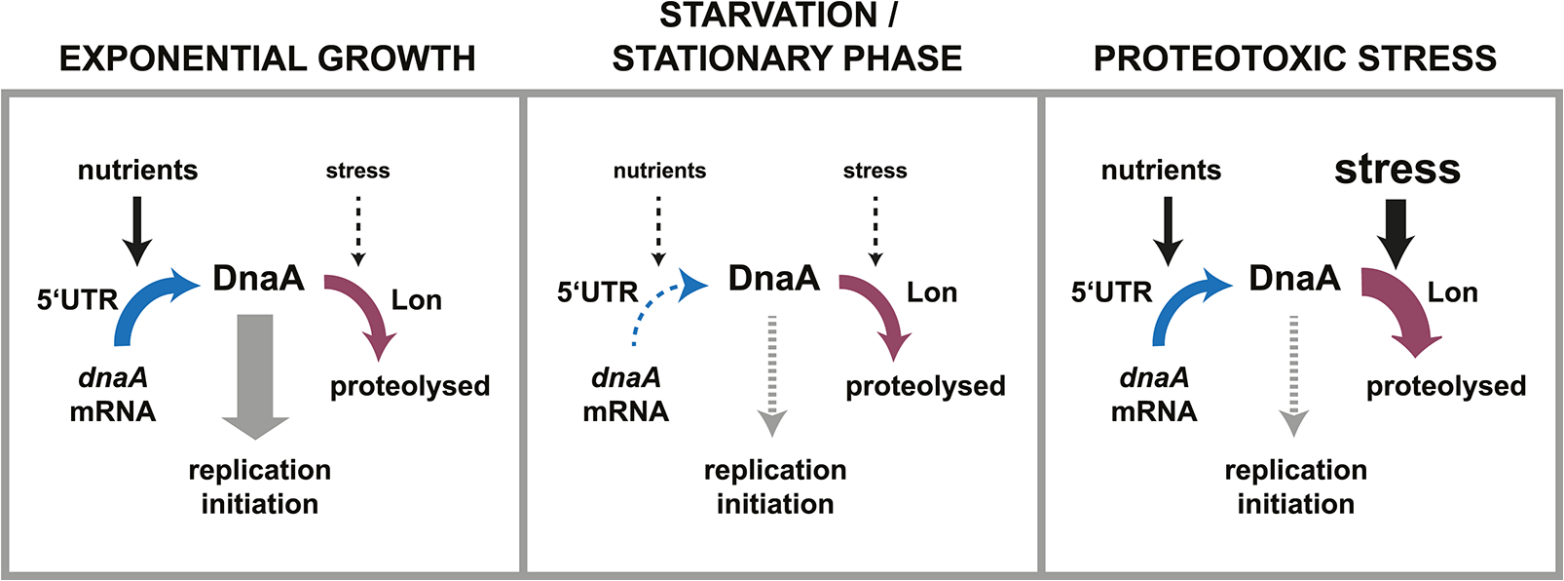
Dynamic control of DnaA abundance and DNA replication in response to environmental inputs. The synthesis and the degradation of DnaA are both subject to control mechanisms that respond to environmental changes. Changes in nutrient availability modulate the rate of DnaA synthesis by a mechanism involving the 5'UTR. Changes in the global protein folding state impact the rate of DnaA degradation by the protease Lon. During exponential growth high levels of nutrients promote translation of DnaA. Although DnaA is constantly degraded, the rate of synthesis is high enough to allow for the accumulation of DnaA and DNA replication initiation. In starvation and stationary phase conditions lower amounts of nutrients cause the translation rate of DnaA to decrease. Because DnaA degradation continues at the same rate as in exponential phase, DnaA is rapidly cleared leading to a cessation of DNA replication. In proteotoxic stress conditions, for example chaperone depletion or thermal stress, nutrients are still available and drive DnaA synthesis. However, Lon-mediated DnaA degradation is stimulated in these conditions leading to the clearance of DnaA and a G1-arrest [26].

Our new results demonstrate that DnaA abundance is tightly regulated by two complementary post-transcriptional mechanisms, which adjust the levels of DnaA in response to nutrient depletion. First, decreasing levels of nutrients slow down DnaA synthesis by affecting its rate of translation; second, Lon-dependent degradation allows DnaA concentration to rapidly drop following the changes in translation (Fig 7). Importantly, the rate of degradation is not significantly affected by changes in nutrient availability. This stands in contrast with proteotoxic stress conditions, which were previously shown to induce the transcription of *lon* and to cause an accumulation of unfolded proteins that can directly stimulate Lon activity and DnaA degradation [26] (Fig 7). Although the rate of DnaA proteolysis does not change upon nutrient exhaustion, a fast constitutive rate of proteolysis is still critical for adjusting the level of DnaA in this condition. Cells containing a deletion of *lon* were unable to clear DnaA and had severe cell cycle defects. Likely, DnaA has evolved a relatively short half-life to allow dynamic changes in its abundance upon environmental inputs through the modulation of the rate of synthesis. Previous proteome-wide studies showed that only a minority of proteins (approx. 4% in *C*. *crescentus*) are proteolytically unstable [45], many of which have important regulatory functions, including CtrA, SciP, FtsZ, CcrM and GcrA [45–47]. In other bacteria, well-studied examples of regulatory proteins with short half-lives include the alternative sigma factors σ^32^ and σ^S^ [48].

Intriguingly, the nutrient-dependent regulation of DnaA synthesis does not act at the level of transcription but instead at the post-transcriptional level by a mechanism involving the 5´UTR of the *dnaA* transcript. A previous study showed that this 5´UTR had a repressing effect on *dnaA* expression during exponential growth [39]. However, a physiological role has not been elucidated until now. Our new data show that the 5´UTR ensures the downregulation of DnaA synthesis in response to nutrient exhaustion. We hypothesize that a small non-coding RNA or a metabolite, produced in a nutrient-dependent manner, may bind to this leader and thereby induce changes in the mRNA secondary structure, which in turn make ribosome binding and translation either more or less efficient depending on nutrient conditions. Alternatively, a regulatory protein might associate with *dnaA* mRNA and affect translational efficiency in a nutrient-dependent manner. In particular, when post-transcriptional control is paired with a short half-life of the target protein, as demonstrated here, the regulation of protein abundance is rapid and precise. We propose that such dynamic control of protein abundance could also be utilized for the better design and construction of synthetic circuits, which so far mainly depend on transcriptional mechanisms [49].

### DnaA abundance is regulated upon stationary phase entry and during carbon starvation

Our study investigated the dynamics of DnaA production and degradation under two conditions: at the entry to stationary phase of cultures grown in complex medium and upon exhaustion of the carbon source glucose in cells grown in minimal medium. In both conditions DnaA synthesis was controlled post-transcriptionally in response to nutrient exhaustion and Lon-mediated proteolysis was required to eliminate the protein. Nevertheless, there are also differences between these conditions. In carbon starvation conditions, the inhibition of DNA replication initiation might also depend on upregulation of CtrA, which directly silences the origin of replication [14], reinforcing previous models that a reciprocal regulation of CtrA and DnaA ensures a coordinated cell cycle block upon growth arrest. Consistent with previous results [25], our data demonstrate that SpoT plays a role in controlling CtrA abundance. Additionally, our data also now indicate a possible role of Lon in this pathway. How exactly these factors affect CtrA abundance and thereby ensure precise regulation of CtrA abundance in response to changing environmental conditions remains to be studied. In other bacteria DNA replication initiation is likely regulated at the onset of stationary phase and carbon starvation as well. In *E*. *coli* it has been observed that cells grown to stationary phase arrest the cell cycle with two or four whole chromosomes [50], indicating that DNA replication initiation is blocked in this condition. The underlying mechanisms remain unclear [51]. Future studies will help to elucidate if the environmental control of DnaA is conserved among bacteria.

### The regulation of DnaA abundance does not require (p)ppGpp

An earlier study proposed that (p)ppGpp regulates DnaA abundance during carbon starvation [24]. In that previous study, synchronized swarmer cells were transferred to M2 minimal medium without any carbon source. In contrast, we investigated DnaA levels and stability in mixed cultures during a less abrupt nutrient exhaustion, which likely better represents the situation in natural environments. We found that the regulation of DnaA abundance at the onset of nutrient exhaustion was not affected in strains which are unable to produce (p)ppGpp, indicating that this signaling molecule is not required for DnaA proteolysis and starvation-induced elimination of DnaA under the conditions tested. In agreement with our data, another recently published study showed that the artificial overproduction of (p)ppGpp does not impact DnaA stability [31]; prolonged (p)ppGpp overproduction affected DnaA synthesis only moderately and indirectly [31]. Although Δ*spoT* cells were still able to clear DnaA at the onset of stationary phase or starvation, we observed deficiencies in arresting cell growth and the cell cycle. The inability of Δ*spoT* cells to arrest the cell cycle might stem from a misregulation of CtrA under starvation conditions. Alternatively, or in addition, other cell cycle processes or replication proteins might be affected by (p)ppGpp. In *B*. *subtilis* and *E*. *coli*, (p)ppGpp is known to affect DNA replication elongation by directly inhibiting DNA primase [52,53].

### Arresting growth and DNA replication as a survival mechanism

Altogether, our results highlight the importance of tightly regulating DNA replication at the onset of adverse conditions demanding growth arrest. The modulation of growth and proliferation is well known to affect bacterial fitness and survival. For instance, entering a non-growing and non-proliferating state has been demonstrated to enhance bacterial drug tolerance and intracellular persistence of pathogenic bacteria [54]. Understanding the regulation of fundamental processes like DNA replication under conditions that require growth arrest is thus important for developing strategies for bacterial growth control.

## Materials and Methods

### Growth conditions

Wild type *C*. *crescentus* NA100 and its mutant derivatives were grown in PYE (complex medium), M2G medium (minimal medium containing 0.2% glucose), M2G_1/10_ medium (minimal medium containing 0.02% glucose) or in M2G with varying amounts of peptone and yeast extract as indicated in Fig 5. When necessary, growth medium was supplemented with 0.3% xylose, 0.2% glucose, 3% sucrose or 1 mM IPTG. For addition of nutrients to a stationary phase grown culture, 10x or 50x stock solutions of PYE were prepared and added as 1:5 or 1:10 dilutions to the stationary-phase grown culture. Note, that the 50x stock contained only the nutrient ingredients (peptone and yeast extract) of PYE medium, but not the salts (MgSO_4_, CaCl_2_). Cultures were grown at 30°C at 200 rpm. Antibiotics were added in the following concentrations as needed for solid and liquid media, respectively: oxytetracycline (2 μg ml^−1^ or 1 μg ml^−1^), kanamycin (25 μg ml^−1^ or 5 μg ml^−1^), chloramphenicol (1 μg ml^−1^ or 2 μg ml^−1^) or spectinomycin (200 μg ml^−1^ or 25 μg ml^−1^). *E*. *coli* strains were routinely grown in LB medium at 37°C, supplemented with chloramphenicol (30 μg ml^−1^ or 20 μg ml^−1^), kanamycin (50 μg ml^−1^ or 30 μg ml^−1^), oxytetracycline (12 μg ml^−1^), or spectinomycin (50 μg ml^−1^) as required.

### Strain construction

Strains used in this study are listed in S1 Table. Deletions of *spoT* and *spoT/ppk1* in strains ML2389 and ML2390 were created by using the two-step recombination procedure [55]. To generate the *spoT* deletion, plasmid pNPTS-*spoT* was introduced into *C*. *crescentus* CB15N by electroporation. Clones that had integrated the vector at the *spoT* locus were selected on PYE plates containing kanamycin. A second recombination step was performed to select for plasmid excision. Single colonies of the first integrants were grown overnight in PYE without kanamycin. After overnight growth, 1 μl was plated for counter-selection on PYE containing sucrose. Sucrose resistant clones were restreaked to test for loss of kanamycin resistance and hence plasmid excision. The resulting clones have either regenerated the wild-type allele or generated the desired in-frame deletion. To distinguish between the two outcomes, PCR was performed to verify deletion of the *spoT* gene. To generate the *spoT/ppk1* strain, the same two-step recombination procedure was performed, except plasmid pNPTS-*ppk1* was introduced into the ML2389 (∆*spoT*) background.

To generate strain KJ743 plasmid pNPTS-*lon*::*tet* ^*r*^ was introduced into strain KJ300 by electroporation. Integrants were selected on plates containing tetracycline and kanamycin. A second recombination step was performed for plasmid excision. PCR and Western blotting were performed to confirm the deletion of the *lon* gene.

Strains KJ741 and KJ742 were generated by electroporating plasmid pRVYFPC-5:*P*_*van*_ *-dnaN-YFP*::*tet* ^*r*^ into strains CB15N or LS2382, respectively.

Strain ML2000 was generated by introducing a *P*_*lacI*_-*lacI* cassette 73 bp upstream of the *hfaA* promoter using the two-step recombination procedure outlined above. Next, 400 bp upstream of *dnaA* was replaced with the 122 bp *P*_*lac*_ promoter also using two-step recombination. Dependence of *dnaA* expression on IPTG was then confirmed by growing the strain in PYE lacking IPTG, verifying replication arrest by flow cytometry, and then verifying cellular filamentation by phase microscopy.

Strains KJ729, KJ730 and KJ731 were generated by electroporating plasmids pCT133-*P*_*dnaA*_ *-UTR*_*dnaA*_ *-dnaA*, pCT133-*P*_*dnaA*_ *-ΔUTR*_*dnaA*_ *-dnaA* and pCT133-*P*_*lac*_ *-UTR*_*dnaA*_ *-dnaA* into strain GM2471.

### Plasmid construction

#### pNPTS-*spoT*

600 bp upstream and 600 bp downstream of the *spoT* coding sequence were cloned into the HindIII and EcoRI sites of pNPTS138 using Sequence and Ligation Independent Cloning (SLIC). The first and last six codons of *spoT* were left intact to prevent polar effects.

#### pNPTS-*ppk1*

901 bp upstream and 846 bp downstream of the *ppk1* coding sequence were cloned into the HindIII and EcoRI sites of pNPTS138 using SLIC. The first five and last eight codons of *ppk1* were left intact to prevent polar effects.

#### pNPTS-*P*_*lac*_ *-dnaA*

Using SLIC, the following DNA sequences were assembled into the HindIII and EcoRI sites of the pNPTS138 vector: (1) 511 bp upstream of the *dnaA* promoter (defined here as the intergenic region between Cog-RpsT and *dnaA* start codon), (2) 122 bp P_lac_ promoter from pEXT20, and (3) 511 bp of the 5’ end of the *dnaA* coding region.

#### pNPTS-*P*_*lacI*_ *-lacI*

Using SLIC, the following DNA sequences were assembled into the EcoRI and XmaI sites of the pNPTS138 vector: (1) 530 bp upstream of position -73 in the *hfaA* promoter, (2) 1196 bp *P*_*lacI*_ *-lacI* fragment from the vector pEXT20, and (3) 517 bp downstream of position -73 in the *hfaA* promoter.

#### pNPTS-*lon*::*tet* ^*r*^

Using Gibson Assembly a tetracycline resistance cassette was inserted between 609 bp upstream and 601 bp downstream of the *lon* coding sequence of the amplified plasmid pNPTS-*lon*. The first five and last nine codons of *lon* were left intact to prevent polar effects.

#### pENTR-*P*_*dnaA*_ *-UTR*_*dnaA*_ *-dnaA*

The *dnaA* coding region plus 400 bp upstream were cloned into the EcoRI and XmaI sites of pNPTS138 using traditional restriction enzyme based methods.

#### pENTR-*P*_*dnaA*_ *-ΔUTR*_*dnaA*_ *-dnaA*

Using SLIC, the following DNA sequences were assembled into the EcoRI and XmaI sites of the pNPTS138 vector: (1) 245 bp promoter fragment missing the *dnaA* 5’ UTR (positions -400 to -156 from start codon), and (2) 1488 bp fragment containing positions -15 (from start codon) to *dnaA* stop codon.

#### pENTR-*P*_*lacI*_ *-lacI-P*_*lac*_ *-UTR*_*dnaA*_ *-dnaA*

Using SLIC, the following DNA sequences were assembled into the EcoRI and XmaI sites of the pNPTS138 vector: (1) 1287 bp *P*_*lacI*_ *-lacI-P*_*lac*_ fragment from pEXT20, and (2) 1628 bp UTR_dnaA_-dnaA fragment (position -155 from start codon to *dnaA* stop codon).

Recombination of these three pENTR plasmids with the destination vector pCT133 using the Gateway LR clonase resulted in pCT133-*P*_*dnaA*_ *-UTR*_*dnaA*_ *-dnaA*, pCT133-*P*_*dnaA*_ *-ΔUTR*_*dnaA*_ *-dnaA* and pCT133-*P*_*lac*_ *-UTR*_*dnaA*_ *-dnaA*.

### Flow cytometry

Samples from *C*. *crescentus* cultures grown in the appropriate conditions were fixed in 70% ethanol. Fixed cells were pelleted at 4000 rpm, resuspended in 50 mM sodium citrate buffer containing 2 μg/ml RNase and incubated at 50°C for 4 hrs or overnight to digest RNA. Samples were diluted and stained with 2.5 μM SYTOX green before being analyzed by flow cytometry using a BD LSRII or a LSRFortessa flow cytometer (BD Biosciences). Flow cytometry histograms were processed with FlowJo software. To quantify the number of cells in G1 phase (1N), with 2N or with a chromosome content >2N, respectively, we used FlowJo. Flow cytometry profiles within one figure were recorded in the same experiment, on the same day with the same settings. The scales of y- and x-axes of the histograms within one figure panel are identical. Each experiment was repeated independently and representative results are shown.

### Microscopy

Cells were fixed with 0.5% paraformaldehyde, pelleted, and resuspended in an appropriate volume of PBS. Fixed cells were mounted onto PYE 1.2% agarose pads and phase contrast images taken using a T*i* eclipse inverted research microscope (Nikon) with a 100x/1.45 NA objective (Nikon). For the analysis of fluorescently marked origins or DnaN-YFP foci, YFP emission/excitation filters were used. ImageJ and Adobe Photoshop were used for image processing.

### Immunoblotting

Pelleted cells, normalized to the optical density of the culture, were resuspended in 1X SDS sample buffer and heated to 95°C for 10 min. Total protein samples were then subjected to SDS-PAGE for 60 min at 130 V at room temperature on 11% Tris-HCl gels and transferred to PVDF or nitrocellulose membranes. Proteins were detected using primary antibodies against DnaA (Jonas et al. 2011), DnaK, RpoA, CtrA or *E*. *coli* Lon (kindly provided by R.T. Sauer) in appropriate dilutions, and a 1:5000 dilution of secondary HRP-conjugated antibody. The primary antibody against *C*. *crescentus* DnaA was affinity purified to enhance specificity and to prevent cross-reactivity with *C*. *crescentus* Hsp. SuperSignal Femto West (Thermo Scientific) was used as detection reagent. Blots were scanned with a Typhoon scanner (GE Healthcare) or a Chemidoc (Bio-rad) system. Images were processed with Adobe Photoshop, and the relative band intensities quantified with ImageJ software.

### *In vivo* degradation assay

To measure protein degradation *in vivo*, cells were grown under the desired conditions. Protein synthesis was blocked by addition of 100 μg/ml chloramphenicol. Samples were taken every 10 min and snap frozen in liquid nitrogen before being analyzed by Western blotting.

### Mathematical modeling

The effect of varying degradation rates on DnaA abundance was investigated using the following equation:

$$\frac{dP\left(t\right)}{dt}=k_{s}-k_{d}\left(t\right)\;P\left(t\right)$$

where *k*_*s*_ is the rate of protein synthesis (assumed constant) and $k_{d}\left(t\right)=a\;t+\frac{\text{ln}\;2}{23\text{min}}$ is a linearly increasing degradation rate, having a value corresponding to a half-life of 23 min at t = 0 (OD_600_ 0.4) (Fig 3B). As the half-life is always much shorter than the doubling time, we can safely ignore the effects of dilution due to growth (or the lack thereof). To generate the solid red curve in Fig 3C, we choose *a* such the half-life at t = 160min (OD_600_ 1.0) is 20min (Fig 3B). We fixed *k*_*s*_ by assuming that protein levels are in steady state at t = 0, a reasonable assumption during exponential phase growth. The equation was solved using the ode45 solver of MATLAB (The MathWorks Inc.).

To find out how fast degradation at OD_600_ 1.0 would have to be in order to explain the data, we used the MATLAB’s constrained non-linear optimization algorithm, fmincon, to find the values of *k*_*s*_ and *a* that result in the best fit to the observed relative DnaA abundance (Fig 3C, blue line) as measured by relative least square. This best fit is plotted as the dashed red line in Fig 3C. The value of *a* found results in a half-life of 9.1min at t = 160min (OD_600_ 1.0).

In order to estimate the rate of DnaA protein synthesis, we allow *k*_*s*_ to vary with time and write $k_{s}\left(t\right)=\frac{dP\left(t\right)}{dt}+k_{d}\left(t\right)\;P\left(t\right)$. We can then calculate *k*_*s*_(*t*) point-wise by using the measured protein abundance (Fig 3E) and half-lives (Fig 3B). We estimate $\frac{dP\left(t\right)}{dt}$ from a linear fit through the data excluding the overnight time point and take the half-life to be 23 min for the first two time points and 20 min for the last two time points. The resulting (normalized) values of the synthesis rate *k*_*s*_, expressed as a function of OD_600_, are presented in Fig 4A (blue line). We convert this synthesis rate into an estimated translation rate by dividing each time-point by the normalized mRNA abundance, as measured by qPCR (red line).

### DNA microarrays

RNA was collected from bacteria that were grown under the appropriate conditions and extracted using the RNeasy mini kit (Qiagen). The generation of labeled cDNA and hybridization of custom Agilent arrays was performed as earlier described [56].

### Quantitative RT-PCR

RNA was collected from bacteria that were grown under the appropriate conditions as described above. Equal amounts of isolated RNA were reverse transcribed into cDNA using the iScript cDNA synthesis kit (Bio-rad). The cDNA was used as template for the real-time PCR reaction using the iTaq universal SYBR Green Supermix (Bio-rad) and primers as listed in S2 Table. Analysis was performed with a qTower instrument (Analytik Jena) using the standard run mode. For detection of primer dimerization or other artefacts of amplification, a dissociation curve was run immediately after completion of the real-time PCR. Individual gene expression profiles were normalized against 16S RNA, serving as an endogenous control. Relative expression levels were determined with the comparative Ct method. Each qPCR reaction was performed in triplicates. The data shown represent means of at least two independent biological replicates.

## Supporting Information

S1 Fig A *ΔspoT* mutation does not affect the stability of DnaA at the onset of stationary phase. *In vivo* degradation assays showing DnaA stability in wild type (WT) and *ΔspoT* cells. Cells were grown in PYE to an OD_600_ of 1.0 before chloramphenicol was added to shut-off protein synthesis. Remaining DnaA levels were monitored by Western Blotting. Band intensities were quantified (bottom).
(EPS)

S2 Fig Total protein loading control for the *in vivo* degradation assays shown in Fig 3. (A) Samples used in Fig 3A loaded on TGX stain-free precast gels that allow rapid fluorescent detection of proteins. (B) Samples used in Fig 3B loaded on TGX stain-free precast gels. (C) Samples used in Fig 3D loaded on TGX Stain-Free precast gels. In all cases, the fluorescent detection of proteins indicates that comparable amounts of total protein were loaded in each set of samples.
(EPS)

S3 Fig DnaA stability is nearly unaffected at the onset to stationary phase at OD_600_ 1.2. *In vivo* degradation assays showing DnaA stability in wild type cells grown in PYE to an OD_600_ of 0.4 or 1.2, respectively, before chloramphenicol was added to shut-off protein synthesis. Remaining DnaA levels were monitored by Western Blotting. Band intensities were quantified (bottom).
(EPS)

S4 Fig A *P*_*lac*_ *-dnaA* strain allows titratable constitutive expression of *dnaA*. The promoter of *dnaA* and its 5´ untranslated leader region (5'UTR) were replaced by *P*_*lac*_, an IPTG-regulated promoter, and its native leader. Western Blots show that addition of 1 mM IPTG to the growth medium results in constitutive *dnaA* expression with DnaA levels comparable to those seen in wild-type cells grown to exponential phase in PYE medium. Addition of 50 μM IPTG results in DnaA levels comparable to those seen in wild-type cells grown in M2G medium.
(EPS)

S5 Fig Control Western Blots for Fig 3E. Samples used in Fig 3E were subject to Western Blotting and probed with an antibody against DnaK. The level of DnaK is not significantly changed indicating comparable loading of total protein.
(EPS)

S6 Fig Control Western Blots for Fig 4E. Samples used in Fig 4E were subject to Western Blotting and probed with an antibody against DnaK. The level of DnaK is not significantly changed indicating comparable loading of total protein.
(EPS)

S7 Fig Control Western Blots for Fig 5. **(**A) Samples used in Fig 5A were subjected to Western Blotting and probed with an antibody against Lon. (B) Samples used in Fig 5B were subjected to Western Blotting and probed with an antibody against DnaK. (C) Samples used in Fig 5D were subjected to Western Blotting and probed with an antibody against DnaK. (D) Samples used in Fig 5E were subjected to Western Blotting and probed with an antibody against DnaK. (E) Samples used in Fig 5F were subjected to Western Blotting and probed with an antibody against DnaK. In all cases, the level of DnaK is not significantly changed indicating comparable loading of total protein.
(EPS)

S8 Fig Effects on DNA replication after nutrient addition to cultures of stationary phase-grown or carbon-starved cells. (A) Changes in DNA content after nutrient addition to a culture grown in stationary phase (see also Fig 5D). A culture was grown for 2 hours in stationary phase (at OD_600_ 1.5) before 5x PYE (final concentration) was added (+ 5x PYE). As controls, one subculture was kept in stationary phase for the rest of the experiment (no addition) and another culture was backdiluted (1:10) into fresh PYE medium (backdilution). DNA content was measured by flow cytometry at the indicated time points. (B) Changes in DNA content after glucose addition to a carbon starved culture (see also Fig 5E). A culture grown in M2G was shifted to M2 medium containing 0.02% glucose to induce carbon starvation. Two hours after the resulting growth arrest the culture was split into two subcultures at t = 0. One of them remained untreated (no addition), the other culture was supplemented with 0.2% glucose (glucose addition). A third culture was grown in M2G medium throughout the experiment (M2G). DNA content was measured by flow cytometry at the indicated time points.
(EPS)

S9 Fig Carbon starvation does not activate the degradation of DnaA. *In vivo* degradation assays showing DnaA stability in wild type cells before and after glucose exhaustion. Cells were shifted from M2G to M2 medium containing 0.02% glucose to deplete glucose. DnaA degradation was monitored at t = 0 hr (before carbon exhaustion) or t = 4 hrs (at entry of growth arrest) by synthesis shut-down assays and Western Blotting. Band intensities were quantified (bottom).
(EPS)

S10 Fig Control Western Blots for Fig 6C. The same samples as used in Fig 6C were used for Western Blotting and probed with an antibody against DnaK. The level of DnaK is not significantly changed indicating equal loading.
(EPS)

S1 Table Strains and plasmids used in this study. (DOCX)

S2 Table Sequences of the qPCR primers used in this study. (DOCX)

S3 Table Microarray gene expression data.</ SI_Caption>
(XLSX)
