# Supplementary material for: Nutritional Control of DNA Replication Initiation through the Proteolysis and Regulated Translation of DnaA
Source: PLoS Genet. 2015 Jul 2;11(7):e1005342. doi: 10.1371/journal.pgen.1005342 (PMC4489657; doi:10.1371/journal.pgen.1005342)
Supplement: S1 Table — (DOCX) [file pgen.1005342.s011.docx]

**S1 Table. Strains and plasmids used in this study.**

| **Strains** | **Description** | **Reference** |
| --- | --- | --- |
| ***C. crescentus*** |  |  |
| CB15N | Synchronizable derivative of wild-type CB15 | [[1](#_ENREF_1)] |
| LS2382 | CB15N Δ*lon* (*spec^r^*) | [[2](#_ENREF_2)] |
| ML2389 | CB15N Δ*spoT* | This study |
| ML2390 | CB15N Δ*spoT* Δ*ppk1* | This study |
| SG400 | *P_xyl_-dnaK-dnaJ (spec^r^)* | [[3](#_ENREF_3)] |
| ML2000 (alt. KJ690) | *P_lac_-dnaA* (native *dnaA* locus), *P_lacI_-lacI* (*hfa* locus) | This study |
| GM2471 | CB15N *dnaA::Ω(spec^r^)*, *P_xyl_::dnaA(kan^r^)* | [[4](#_ENREF_4)] |
| KJ729 | GM2471 + pCT133-*P_dnaA_-UTR-dnaA* | This study |
| KJ730 | GM2471 + pCT133-*P_dnaA_-ΔUTR-dnaA* | This study |
| KJ731 | GM2471 + pCT133-*P_lacI_-lacI-P_lac_-UTR-dnaA* | This study |
| KJ741 | CB15N + pRVYFPC-5:*P_van_-dnaN-YFP::tet^r^* | This study |
| KJ742 | LS2382 + pRVYFPC-5:*P_van_-dnaN-YFP::tet^r^* | This study |
| KJ300 | *cc0006::(tetO)_n_ (gent^r^), P_van_****:****tetR-eyfp* | [[5](#_ENREF_5)] |
| KJ743 | *cc0006::(tetO)_n_ (gent^r^), P_van_****:****tetR-eyfp,* Δ*lon* (*tet^r^*) | This study |
| ***E. coli*** |  |  |
| DH5α | General cloning strain | Invitrogen |
| TOP10 | General cloning strain | Invitrogen |
| **Plasmids** |  |  |
| pNPTS138 | Integration vector | Lab collection |
| pENTR/D-TOPO | ENTRY vector for Gateway cloning system (*kan^r^*) | Invitrogen |
| pCT133 | Destination vector of pMR20, low copy plasmid (*tet^r^*) | Lab collection |
| pNPTS-*spoT* | For generation of a markerless in-frame deletion of *spoT* | This study |
| pNPTS-*ppk1* | For generation of a markerless in-frame deletion of *ppk1* | This study |
| pNPTS-*P_lac_-dnaA* | For generation of a IPTG controllable *dnaA* expression strain | This study |
| pNPTS-*P_lacI_-lacI* | For generation of a IPTG controllable *dnaA* expression strain | This study |
| pENTR-*P_dnaA_-UTR-dnaA* | pENTR containing *dnaA* and 400 bp of its upstream region | This study |
| pENTR-*P_dnaA_-ΔUTR-dnaA* | pENTR containing *dnaA* and the *dnaA* promoter (260 bp), but lacking 140 bp of the 5'UTR | This study |
| pENTR-*P_lacI_-lacI-P_lac_-UTR-dnaA* | pENTR containing the *P_lac_* promoter fused to the 5'UTR of *dnaA* and the *dnaA* gene | This study |
| pCT133-*P_dnaA_-UTR-dnaA* | pCT133 containing *dnaA* and 400 bp of its upstream region | This study |
| pCT133-*P_dnaA_-ΔUTR-dnaA* | pCT133 containing *dnaA* and the *dnaA* promoter (260 bp), but lacking 140 bp of the 5'UTR | This study |
| pCT133-*P_lacI_-lacI-P_lac_-UTR-dnaA* | pCT133 containing the *P_lac_* promoter fused to the 5'UTR of *dnaA* and the *dnaA* gene | This study |
| pRVYFPC-5:*P_van_-dnaN-YFP::tet^r^* | pRVYFPC-5 containing the *dnaN* gene | [[6](#_ENREF_6)] |
| pNPTS-*lon* | For generation of a markerless deletion of *lon* | [[7](#_ENREF_7)] |
| pNPTS-*lon::tet^r^* | For generation of a tet-marked deletion of *lon* | This study |

**S1 Table References**

1. Evinger M, Agabian N (1977) Envelope-associated nucleoid from Caulobacter crescentus stalked and swarmer cells. J Bacteriol 132: 294-301.

2. Wright R, Stephens C, Zweiger G, Shapiro L, Alley MR (1996) Caulobacter Lon protease has a critical role in cell-cycle control of DNA methylation. Genes Dev 10: 1532-1542.

3. da Silva AC, Simao RC, Susin MF, Baldini RL, Avedissian M, et al. (2003) Downregulation of the heat shock response is independent of DnaK and sigma32 levels in Caulobacter crescentus. Mol Microbiol 49: 541-553.

4. Gorbatyuk B, Marczynski GT (2001) Physiological consequences of blocked Caulobacter crescentus dnaA expression, an essential DNA replication gene. Mol Microbiol 40: 485-497.

5. Jonas K, Chen YE, Laub MT (2011) Modularity of the bacterial cell cycle enables independent spatial and temporal control of DNA replication. Curr Biol 21: 1092-1101.

6. Aakre CD, Phung TN, Huang D, Laub MT (2013) A bacterial toxin inhibits DNA replication elongation through a direct interaction with the beta sliding clamp. Mol Cell 52: 617-628.

7. Jonas K, Liu J, Chien P, Laub MT (2013) Proteotoxic stress induces a cell-cycle arrest by stimulating Lon to degrade the replication initiator DnaA. Cell 154: 623-636.
