# Supplementary material for: Nutritional Control of DNA Replication Initiation through the Proteolysis and Regulated Translation of DnaA
Source: PLoS Genet. 2015 Jul 2;11(7):e1005342. doi: 10.1371/journal.pgen.1005342 (PMC4489657; doi:10.1371/journal.pgen.1005342)
Supplement: S2 Table — (DOCX) [file pgen.1005342.s012.docx]

**S2 Table. Sequences of the qPCR primers used in this study.**

| Name | Sequence (5' to 3') |
| --- | --- |
| RT_dnaAFor | CGGACGGTCACTTCAATCCT |
| RT_dnaARev | GCGTTCAGCAAGTGCGTTT |
| RT_16SFor | GGGTTAAGTCCCGCAACGA |
| RT_16SRev | ATGATTAGAGTGCCCAGCCAAA |
| RT_katGFor | CTACATGGCCCCGGAAGAGG |
| RT_katGRev | GTGAACACCCCGTCCTTGGA |
| RT_l13pFor | GTGCTGGGCGGTCGTTTCC |
| RT_l13pRev | GGCTCCGGATGTTCTTGGCG |
